# A heart so black: a case of alkaptonuric ochronosis of the aortic and mitral valves in a female patient with severe aortic valve stenosis and coronary artery disease

**DOI:** 10.1093/jscr/rjae644

**Published:** 2024-10-11

**Authors:** Alex Kamougeros, George Shiakos, Stelios Ioannou, Ioannis Tzanavaros, Zeyad Al-Jazrawi, Beatrice Ioannou

**Affiliations:** Cardiac Innovation Center, Apollonion Hospital, Lefkotheou 20, Nicosia, Nicosia 2054, Cyprus; Cardiac Innovation Center, Apollonion Hospital, Lefkotheou 20, Nicosia, Nicosia 2054, Cyprus; European University Medical School, Nicosia, Cyprus; Cardiac Innovation Center, Apollonion Hospital, Lefkotheou 20, Nicosia, Nicosia 2054, Cyprus; Cardiac Innovation Center, Apollonion Hospital, Lefkotheou 20, Nicosia, Nicosia 2054, Cyprus; University of Nicosia Medical School, Nicosia, Cyprus; Cardiac Innovation Center, Apollonion Hospital, Lefkotheou 20, Nicosia, Nicosia 2054, Cyprus; Cardiac Innovation Center, Apollonion Hospital, Lefkotheou 20, Nicosia, Nicosia 2054, Cyprus

**Keywords:** alkaptonuric ochronosis, aortic valve stenosis, cardiac valve ochronosis

## Abstract

Alkaptonuric ochronosis, characterized by the deposition of homogentisic acid in connective tissues, is commonly linked with alkaptonuria, a rare genetic disorder resulting from homogentisate 1,2-dioxygenase deficiency. Despite its association with alkaptonuria, ochronosis can occur in individuals without a prior diagnosis. This case report discusses a 64-year-old female with severe aortic valve stenosis and coronary artery disease who was found to have ochronotic pigmentation in the aortic and mitral valves, as well as in the aortic root intima and papillary muscles. This case emphasizes the need to consider ochronosis in the differential diagnosis of valvular disease when alkaptonuria is suspected.

## Introduction

Ochronosis is a rare and significant condition resulting from the accumulation of homogentisic acid in connective tissues, leading to distinctive dark pigmentation and tissue degeneration [1]. It is most often associated with alkaptonuria, an autosomal recessive disorder caused by a deficiency in homogentisate 1,2-dioxygenase, an enzyme crucial for breaking down homogentisic acid [2]. This metabolic defect results in the systemic deposition of homogentisic acid in various tissues, including cartilage, skin, and the cardiovascular system.

Ochronotic deposition involving the aortic valve or the coronary arteries is particularly rare, with only a few cases documented in the literature. The rarity of this condition is further heightened by the fact that alkaptonuria itself is a rare disorder, occurring in ~1 in 250 000 to 1 in 1000 000 live births worldwide. Due to its low prevalence, the exact incidence of ochronosis remains unclear, often being discovered incidentally during valve replacement procedures or autopsies.

Although alkaptonuria is the most common cause of ochronosis, the condition can also manifest without a prior diagnosis, complicating clinical evaluation. Cardiac involvement in ochronosis, while uncommon, can lead to significant pathology, particularly affecting the aortic and mitral valves, with serious clinical implications [2]. This case report describes a 64-year-old female with severe aortic stenosis and coronary artery disease who was unexpectedly found to have ochronotic pigmentation during surgery, despite having no known history of alkaptonuria.

## Case report

A 64-year-old female presented with severe aortic valve stenosis and coronary artery disease presenting with easy fatigue on exertion, without a prior diagnosis of alkaptonuria or any metabolic disorders. The patient presented with preserved left ventricular systolic function (ejection fraction 60%), although cardiac imaging revealed abnormalities, including an elevated left ventricular end-diastolic pressure of 20 mmHg, indicating impaired diastolic function. There was no mitral regurgitation, suggesting normal mitral valve function.

Preoperative aortography revealed a normal-sized proximal ascending aorta, but with restricted opening of calcified aortic cusps, consistent with severe aortic stenosis. Mild aortic incompetence was also noted.

Coronary angiography revealed minimal disease in the left main coronary artery, with moderate to significant (60–70%) stenosis in the left anterior descending artery (LAD). The instantaneous wave-free ratio (iFR) of 0.77 confirmed the functional significance of this lesion. The remaining coronary arteries showed signs of atheromatous disease, however without significant stenosis.

**Figure 1:**
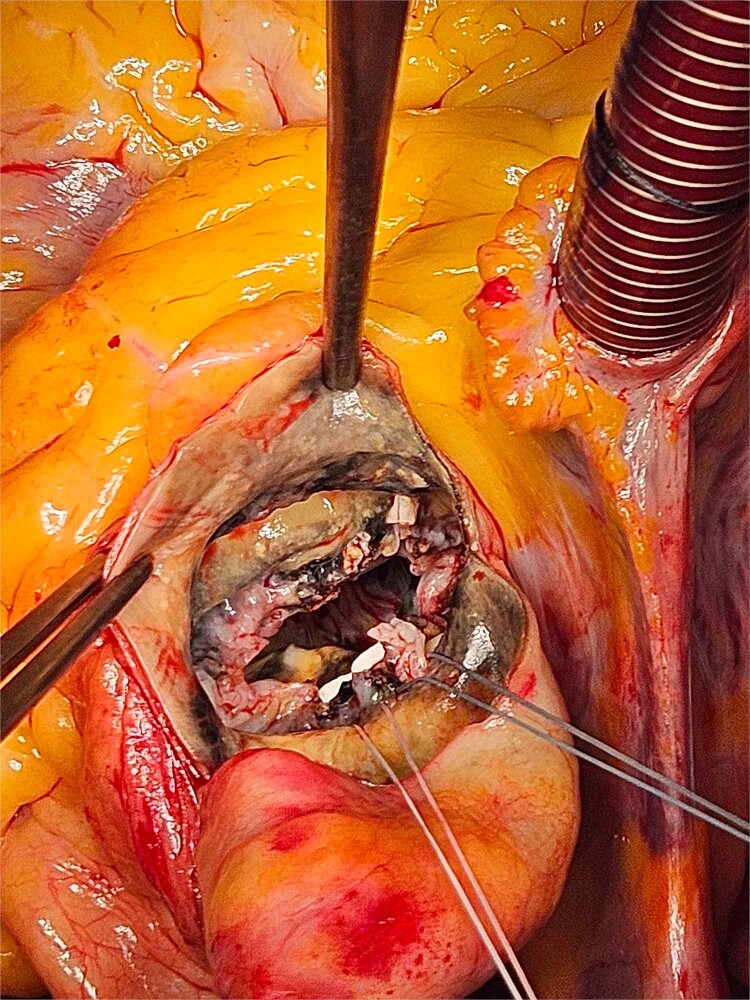
The patchy ochronotic discolouration can be visualized along the aortic root walls and extending below towards the mitral valve.

**Figure 2:**
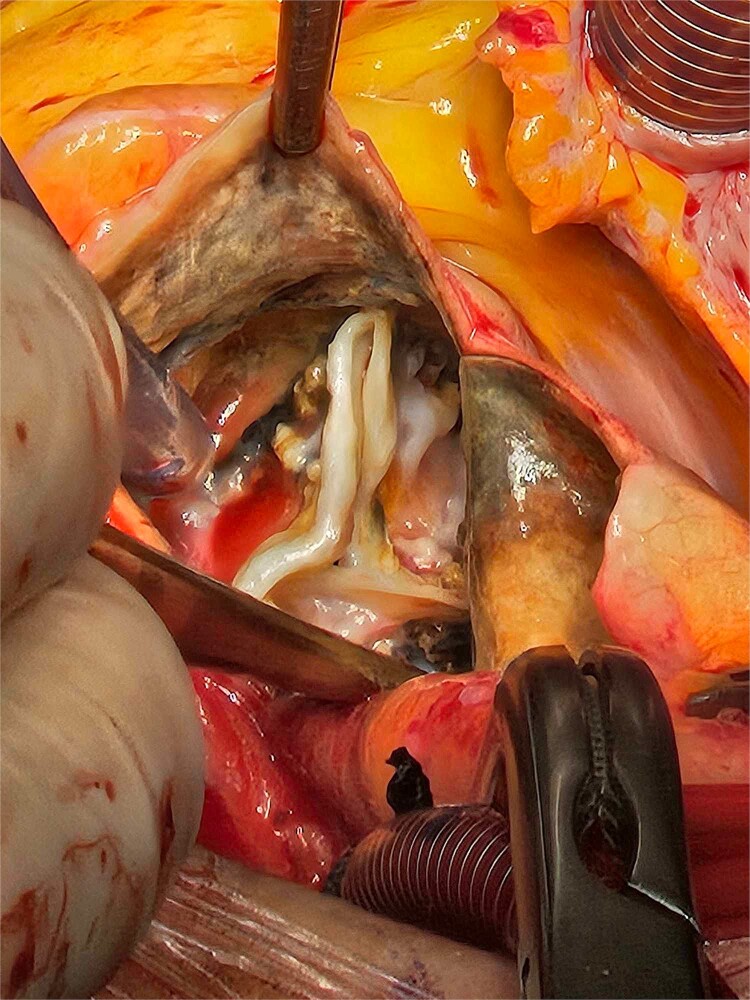
The patchy ochronotic discolouration can be visualized along the aortic root walls, and along the aortic valve leaflets, being more prominent along areas of heavy calcification.

**Figure 3:**
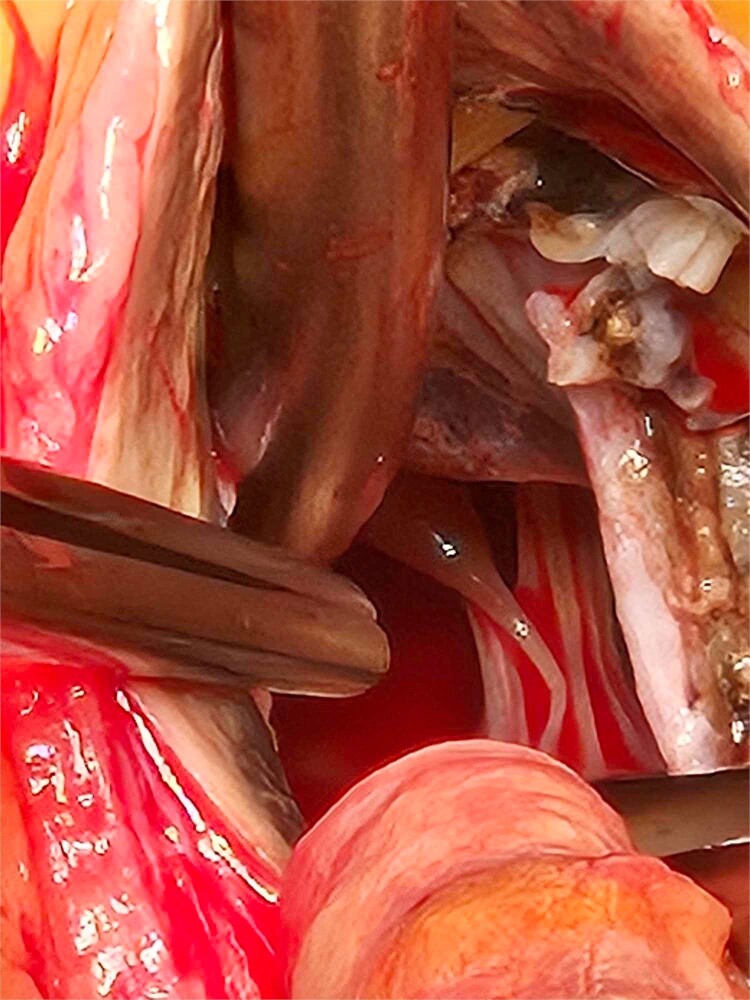
The extent of the ochronotic uptake can be seen extending to the level of the papillary muscles.

Based on these findings, the patient underwent surgical aortic biological valve replacement and coronary artery bypass grafting (CABG) using a left internal mammary artery graft to the LAD. Intraoperatively, unexpected ochronotic pigmentation was observed in the aortic and mitral valve leaflets, papillary muscles, and the intimal layer of the aortic root, presenting as patchy black discoloration (Figs 1–3). Subsequent biochemical analysis confirmed elevated homogentisic acid levels in the urine, consistent with alkaptonuria.

Upon the 7- and 30-day follow-up of the patient post discharge from our center, there were no significant findings indicating complications or deterioration of her condition, with an overall uneventful hospital stay and immediate post-operative period. The patient was duly referred for further investigation to a rheumatology specialist for reference to the finding of alkaptonuria.

## Discussion

Although rare, ochronosis can have significant implications for cardiovascular health. The deposition of homogentisic acid in connective tissues leads to characteristic dark pigmentation and progressive tissue degeneration, particularly affecting the aortic and mitral valves. This case highlights the potential for ochronosis to exacerbate or even cause valvular pathology [3], even in the absence of a prior alkaptonuria diagnosis, which may become evident intraoperatively, as in our presented case.

In this patient, severe aortic stenosis and coronary artery disease were complicated by the presence of ochronosis, which likely contributed to the severity of the valvular disease. Ochronotic pigment deposition can accelerate calcification and fibrosis of the valves, worsening stenosis and impairing cardiac function [4]. The necessity for both CABG and valve replacement in this patient underscores the multidimensional nature of her condition in the context of a rare metabolic disorder such as alkaptonuria.

The incidental finding of tissue ochronosis during surgery emphasizes the need for vigilance in diagnosing rare conditions that may not be evident in preoperative evaluations. Given that alkaptonuria occurs in ~1 in 250 000 to 1 in 1000 000 live births worldwide, clinicians should consider ochronosis in patients with unexplained or severe valvular disease, at least when the patient exhibits further manifestations of a metabolic disorder. Intraoperative findings, combined with specialized biochemical tests, are essential for identifying such rare conditions and for effectively planning successful interventions regarding the alternate manifestations of alkaptonuria in affected patients and to minimize the implications frequently associated with familial correlations of the disease [1, 2].

This case illustrates the importance of considering alkaptonuric ochronosis in the differential diagnosis of valvular and coronary artery disease, especially when relative incidental intraoperative findings arise. The discovery of ochronotic pigmentation during surgery underscores the need for thorough examination and awareness of rare conditions that may not be apparent preoperatively. Recognizing these conditions can significantly impact treatment strategies and patient outcomes, highlighting the need for comprehensive diagnostic approaches in complex cardiac cases, as well as aiding in the improvement of prognostic assessment for such patients.

## Conflict of interest statement

None declared.

## Funding

None declared.
